# Folate-Chitosan Nanoparticles Loaded with Ursolic Acid Confer Anti-Breast Cancer Activities *in vitro* and *in vivo*

**DOI:** 10.1038/srep30782

**Published:** 2016-07-29

**Authors:** Hua Jin, Jiang Pi, Fen Yang, Jinhuan Jiang, Xiaoping Wang, Haihua Bai, Mingtao Shao, Lei Huang, Haiyan Zhu, Peihui Yang, Lihua Li, Ting Li, Jiye Cai, Zheng W. Chen

**Affiliations:** 1 State Key Laboratory of Quality Research in Chinese Medicines, Macau University of Science and Technology, Macau, 999078, China; 2 Department of Microbiology and Immunology, University of Illinois, Chicago 60612, USA; 3 The First Affiliated Hospital of Jinan University, Guangzhou 510632, China; 4 Department of Chemistry, materials science and engineering, Jinan University, Guangzhou 510632, China; 5 Treatment and Research Center of Infectious Diseases, the 302 Hospital of PLA, Beijing, 100039, China

## Abstract

Ursolic acid (UA) has proved to have broad-spectrum anti-tumor effects, but its poor water solubility and incompetent targeting property largely limit its clinical application and efficiency. Here, we synthesized a nanoparticle-based drug carrier composed of chitosan, UA and folate (FA-CS-UA-NPs) and demonstrated that FA-CS-UA-NPs could effectively diminish off-target effects and increase local drug concentrations of UA. Using MCF-7 cells as *in vitro* model for anti-cancer mechanistic studies, we found that FA-CS-UA-NPs could be easily internalized by cancer cells through a folate receptor-mediated endocytic pathway. FA-CS-UA-NPs entered into lysosome, destructed the permeability of lysosomal membrane, and then got released from lysosomes. Subsequently, FA-CS-UA-NPs localized into mitochondria but not nuclei. The prolonged retention of FA-CS-UA-NPs in mitochondria induced overproduction of ROS and destruction of mitochondrial membrane potential, and resulted in the irreversible apoptosis in cancer cells. *In vivo* experiments showed that FA-CS-UA-NPs could significantly reduce breast cancer burden in MCF-7 xenograft mouse model. These results suggested that FA-CS-UA-NPs could further be explored as an anti-cancer drug candidate and that our approach might provide a platform to develop novel anti-cancer drug delivery system.

Cancer remains one of the most devastating diseases threatening public health, causing high mortality worldwide every year. For decades, chemotherapy has served as the preferred treatment. However, conventional chemotherapeutics can’t distinguish cancer cells from normal cells, and inevitably damage healthy cells and tissues with evident toxicity. Therefore, it is of central importance to develop efficacious ant-cancer drugs that selectively target cancer cells with low toxicity.

Ursolic acid (UA) is a triterpenoid compound, which exists extensively in food, medicinal herbs, and other plants. Recently, it has been reported that UA can inhibit the growth and development of prostate cancer, liver cancer, and cervical carcinoma^1^,^2^. Although UA has good anti-cancer activity, clinical application and efficacy of it are still largely limited by its poor water solubility and off-targeting property.

Nanomaterials have recently been emerging as attractive pharmacological vehicles for drug delivery and cancer therapy. The engineered nanomaterials can gain unusual physiochemical characteristics because of their small sizes, surface structure, solubility and shapes. Importantly, nanomaterials can be designed as nanoscale drug carriers to avoid immune clearance by lymphocyte-macrophage system, and therefore allow drugs to efficiently target cancer cells. Over the past decades, inorganic nanoparticles have been explored as drug carriers for new anti-cancer treatments, as nanoparticles can be synthesized to have regular shapes, size, surface chemical and physical properties for better targeting of cancer cells^3^,^4^. However, inorganic nanoparticles can hardly be degraded *in vivo*, leading to maintenances in the body for a long time. Moreover, some inorganic nanoparticles have the intrinsic capability to form large aggregates^5^, which result in harmful metabolites and chronic toxicity due to redistribution or accumulation in vital organs in the body^6^. Recently, Chan *et al*.^7^ described the use of DNA to control the biological delivery and elimination of inorganic nanoparticles by organizing them into colloidal superstructures, highlighting new directions in the design of biodegradable and multifunctional nanomedicine.

While nanoparticles have been used to load UA, some methods for synthesis of UA-loaded nanoparticle exhibit low reproducibility^8^ or require tough reaction condition (supercritical anti-solvent process)^9^. Notably, Xiang *et al*.^10^ encapsulated UA in folate(FA)-targeted liposomes and such formulation largely enhanced the solubility and bioavailability of UA. Sun *et al*.^11^ developed stably-controlled UA-loaded nanoparticles using amphiphilic mPEG–PCL block copolymers, and demonstrated that such a nano-drug delivery system improved the anti-cancer efficiency of UA leading to more cell apoptosis through stronger inhibition of COX-2 and of caspase-3.

We recently made efforts to design novel biodegradable nanoparticles for loading UA and for improving UA solubility/targeting and anti-cancer efficiency. We employed two innovative approaches in the production of potentially useful UA-loaded nanoparticles. First, we used chitosan (CS) for synthesis of UA-loaded nanoparticles. CS is a linear cationic polysaccharide obtained from natural sources of chitin, such as crabs, krill, and shrimps, which has been regarded as a good candidate for the drug delivery system^12^,^13^ owing to its excellent biocompatibility, biodegradability and nontoxicity. It actually can protect the drugs from the surrounding medium or low PH condition under the digestive system through encapsulating active drugs or components in CS polymer micells. Second, we formulated CS, folate(FA), and UA to develop biodegradable nanoparticles (FA-CS-UA-NPs), in which folate molecules were enriched on surface while UAs were encapsulated. Such potentially useful FA-CS-UA-NPs could specifically bind and target folate receptor expressed on cancer cells for delivering UA in the UA-loaded nanoparticles into cells.

We then conducted *in vitro* and *in vivo* studies to evaluate novel FA-CS-UA-NPs for the ability to enhance the anti-breast cancer activities and cancer-targeted features or mechanisms. We demonstrated that FA-CS-UA-NPs internalized into cancer cells via folate receptor-mediated pathway and then induced apoptosis in MCF-7 cells through a mitochondria-dependent pathway. Notably, FA-CS-UA-NPs NPs could significantly reduce breast cancer burden in xenograft mouse model. Thus, our approach could provide a platform to design/develop anti-cancer nano-delivery system especially for drugs with poor water solubility.

## Results

### Characteristics of FA-CS-UA-NPs

**Figure 1:**
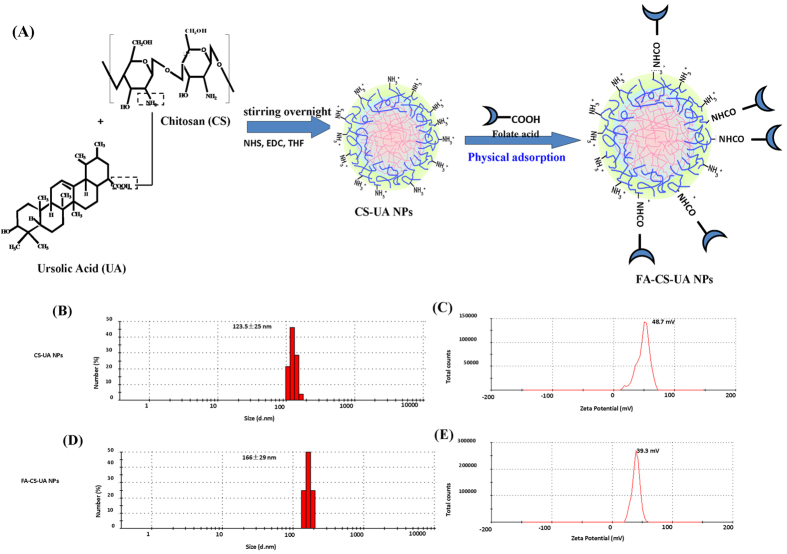
The synthesis and characteristics of folate-coated chitosan nanoparticles loaded UA (FA-CS-UA-NPs). (**A**) Illustration of preparation and modification of CS-UA-NPs. (**B,D**) and (**C,E**) showed mean sizes and zeta potentials of CS-UA-NPs and FA- CS-UA-NPs, respectively.

The method for synthesis of FA-CS-UA-NPs was straight forward, and undergoing the mild reaction condition. Figure 1A showed the synthesis process of FA-CS-UA NPs. As shown in Fig. 1B,D, sizes of CS-UA-NPs ranged about 100~180 nm, with a mean of 122 nm, while the mean size increased to about 160 nm after folate was conjugated on the surface. Figure S1 (Supporting Information) showed the FA-CS-UA-NPs were in irregular shapes.

Since the electric charges on nanoparticle surface play important roles in physical stability and biocompatibility of nanoparticle-based suspensions, we sought to examine zeta potential of FA-CS-UA-NPs. As shown in Fig. 1C,E, the zeta potential of CS-UA-NPs and FA-CS-UA-NPs was +48.7 and +39.3 mV, respectively. The positive charges of FA-CS-UA-NPs implicated that the amino groups of chitosan were presented on the surface of nanoparticles, and the value of zeta potential (>25.0 mv) implicated that the NPs suspensions were stable and not easy to aggregate.

The HPLC results showed that the drug (UA) loading rate was about 50%.

### Cellular uptake of FA-CS-UA-NPs

Many studies^14^,^15^ have proved that uptake and accumulation of nanomaterials in cells is one of the main factors to generate cytotoxicity. To quantify the uptake level of FA-CS-UA-NPs by cancer cells, fluorescence dye rhodamine-B was encapsulated in the FA-CS-UA-NPs, and the cellular uptake level of FA-CS-UA-NPs was determined by measuring the mean fluorescence intensity (MFI) in cells.

The images obtained from FITC channel by confocal microscopy showed the red fluorescence of the rhodamine B- encapsulated nanoparticles. As shown in Figure S2, increased MFIs in MCF-7 cells were in a dose-dependent manner after 3 hour-treatment with FA- CS-UA-NPs. Most of FA- CS-UA-NPs entered into cells. When MCF-7 cells were incubated with 40 μg/ml FA-CS-UA-NPs for different periods, rhodamine B- encapsulated nanoparticles were still seen in cancer cells 96 hours (Figure S3).

Cellular uptake and endocytic pathways of nanomaterials can critically affect the delivery efficiency and bioavailability of the nano-carrier. There are three main ways of internalizing NPs by cells, i.e. endocytosis, fluid phase endocytosis, and receptor-mediated endocytosis^16^. The HPLC results showed that the concentration of UA in MCF-7 cells after the 24-hr treatment with the same concentration (2.0 μg/ml) of UA and FA-CS-UA-NPs were 1.1 and 1.4 μg/ml, respectively. These results suggested that FA-CS-UA-NPs were much easily internalized by MCF-7 cells.

Given the conjugation of folate on the surface of CS-UA-NPs, we assumed that FA-UA-CS-NPs internalized into cells through folate receptor-mediated endocytosis. To test this hypothesis, MCF-7 cells expressing folate receptors(folate receptor+) and Colo205 cells expressing no or very low-level folate receptors(folate receptor−) were selected as models, respectively. Fluorescence-based flow cytometry was used to quantitatively measure the uptake level of FA-UA-CS-NPs. To facilitate examination of folate receptor-mediated enodytosis, cells were pretreated with 0.1 mM folate for 1 h to make sure that the folate receptors on cell membrane was blocked or ligated.

**Figure 2:**
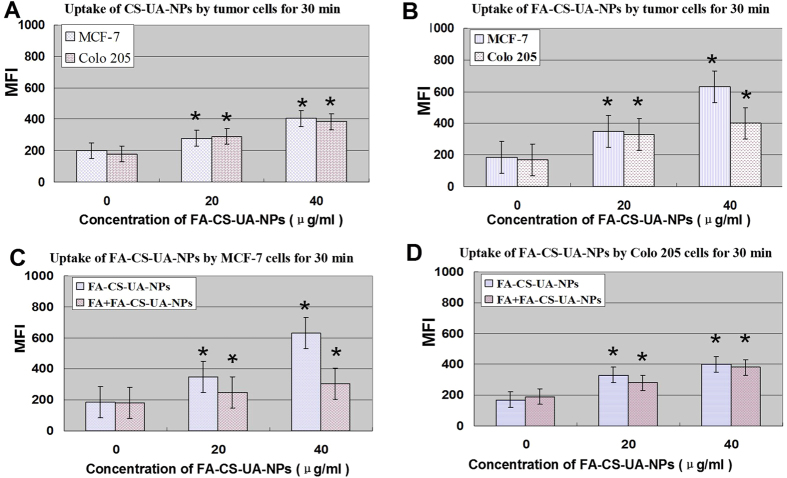
Effects of folate receptors on cellular uptake of FA-CS-UA-NPs. The cellular uptake of CS-UA-NPs (**A**) and FA-CS-UA-NPs (**B–D**) by MCF-7 cells and Colo205 cells in the presence and absence 0.1 mM of folate pretreatment for 30 min (**C,D**), respectively. The resulting MFI of RhB loaded in NPs was indicator of the uptake of NPs. ^*^p < 0.05 for comparisons between test and control groups in all sub-figures; in Fig. 2C, p < 0.05 is also seen for comparisons between FA + FA-CS-UA-NPs and FA-CS-UA-NPs groups.

When these two kinds of tumor cells were incubated with non-folate conjugation NPs (CS-UA-NPs) for 30 min at 37 °C, there were no apparent differences in MFI of CS-UA-NPs between MCF-7 and Colo205 cells (Fig. 2A). In fact, when these two kinds of tumor cells were incubated with folate-conjugated NPs (FA-CS-UA-NPs), there were significant increases in MFI in folate receptor + MCF-7 cells but not folate receptor- Colo205 cells (Fig. 2B), indicating that FA-CS-UA-NPs was more readily uptaken by folate receptor + MCF-7 cancer cells. Moreover, MFI of FA-CS-UA-NPs in control MCF-7 cells not pretreated with folate was remarkably higher than that of MCF-7 cells pretreated with folate (Fig. 2C), suggesting that the cellular uptake of FA-CS-UA-NPs was decreased by blocking folate receptors on cell membrane. Consistently, when folate receptor- Colo205 cells were pretreated with free folate molecules and then cultured with FA-CS-UA-NPs, there were no remarkable changes in MFI in colon cells regardless of folate pretreatment (Fig. 2D).

It was likely that when folate receptor + MCF-7 cells were pretreated with free folate molecules, the binding sites of folate receptors on cellular membrane were blocked, leading to significant decreases in the folate receptor-mediated endocytosis of FA-CS-UA-NPs (Fig. 2C). On contrary, folate receptor- Colo205 cells exhibited no significant changes in the cellular uptake of FA-CS-UA-NPs regardless of folate pretreatment, further suggesting that FA-CS-UA-NPs were internalized by cancer cells via a folate receptor-mediated endocytosis pathway.

The quantitative analysis also suggested that FA-UA-CS-NPs were internalized distinctly by two types of cancer cells. Figure S4A also showed that when the concentration of FA-UA-CS-NPs was lower than 40 μg/ml, the MFI in two kinds of cells was almost similar. However, when the concentration increased to 80 μg/ml, MFI of MCF-7 cell was significantly higher than that of Colo205 cells. Since folate molecules were enriched on the surface, FA-CS-UA-NPs appeared to readily target MCF-7 cancer cells expressing high-level folate receptors. The results supported the point that the cellular uptake was facilitated by a folate receptor-mediated endocytosis pathway.

Figure S4B showed the changes in MFI in cells incubated with the same concentration (40 μg/ml) of FA-UA-CS-NPs at 37 °C for different time (0.5, 2, 3 h, respectively). Increased MFI was seen in cells when the incubation time increases. On the other hand, when the cells were incubated with the same concentration of FA-CS-UA-NPs (40 μg/ml) for the same period (30 min) in different temperatures, MFI at 37 °C was significantly higher than that at 4 °C (Figure S4A,B). These results implied that the cellular uptake of FA-UA-CS-NPs in cancer cells was also in temperature-dependent and time-dependent manner.

### Localization of FA-CS-UA-NPs in cells

**Figure 3:**
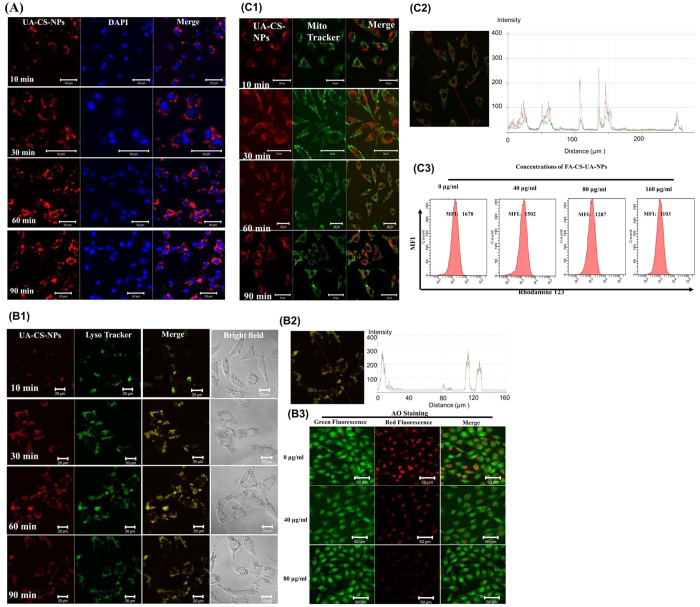
Intracellular trafficking of rhodamine B-loaded FA-CS-UA-NPs after internalized by MCF-7 cells. (**A**) The FA-CS-UA-NPs (left column), cellular nuclei (middle column), and the merged images demonstrated that nanoparticles (red fluorescence) circumvented around the nuclei (blue fluorescence), but no overlay of the two kinds of fluorescence was observed, suggesting that nuclei were not the cellular targets of FA-CS-UA-NPs. Figure B1–B3 showed the effects of FA-UA-CS-NPs on cellular lysosome: (B1,B2) revealed the colocalization of FA-UA-CS-NPs (red) and lysosome (green) in MCF-7 cells after exposure to 20 μg/ml of FA-CS-UA-NPs for different periods of time; (B3) displayed integrity of the lysosomal membrane (AO staining) in MCF-7 cells after cultured with FA-CS-UA-NPs for 6 h, respectively. Scale bar: 20 μm. Figure C1–C3 showed effects of FA-UA-CS-NPs on mitochondria: (C1) revealed colocalization of FA-UA-CS-NPs (red) and mitochondria (green) in MCF-7 cells after exposure to 40 μg/ml FA- CS-UA- NPs for different periods; (C2) showed the profile indicated the colocalization of FA-UA-CS-NPs and mitochondria; (C3) compared ΔΨ in MCF-7 cells after cultured with FA-CS-UA-NPs for 6 h, respectively. Scale bar: 50 μm.

We then examined the fate of FA-CS-UA-NPs after internalization into cells. To this end, we selected MCF-7 cells as a model to determine the localization of FA-CS-UA-NPs in cells. As shown in Fig. 3A, the red fluorescence represented rhodamine B-associated FA-CS-UA-NPs internalized in MCF-7 cells and the blue fluorescence reflected the nuclei specific marker DAPI. The red fluorescence of FA-CS-UA-NPs was increased in a time-dependent manner, and most of FA-CS-UA-NPs entered into the cytoplasm and circumvented around the nuclei, but no overlay of red and blue color was observed. The results suggested nuclei were not the targets of FA-CS-UA-NPs, and that FA-CS-UA-NPs would not cause genotoxicity in cells during the early stage of FA-CS-UA-NPs incubation.

### Effects of FA-UA-CS-NPs on lysosomes and lysosomal membrane integrity

It has been reported that the intracellular localization of nanomaterials closely involved lysosomes and mitochondria^17^ after entry into cells. Here, the distribution of FA-UA-CS-NPs in MCF-7 cells was examined using LysoTracker as a marker of lysosome to investigate the relationship between lysosome locations and FA-UA-CS-NPs. Figure 3B1 showed the co-localization of FA-UA-CS-NPs and lysosomes in MCF-7 cells, and the amounts of FA-UA-CS-NPs located in the lysosomes were increased in a time-dependent manner. The cross line analysis of fluorescence image (Fig. 3B2) also showed the red and green fluorescence were almost entirely coincident.

The integrity of lysosome membrane is important indicator to reflect lysosomal physiological functions. It is well accepted that the long-term accumulation and retention of nanoparticles in lysosomes can influence the lysosomal membrane integrity and further impact their functions. Here, the metachromatic fluorescent cationic dye acridine orange (AO) was used as a probe to detect changes in the integrity of lysosomal membrane induced by as-synthesized nanoparticles. AO could emit strong red light when it accumulates in lysosomes at high concentration, and it will emit green light with weak red fluorescence when it’s not concentrated in lysosomes. Nanomaterials such as carbon nanotube, cationic nanoparticles, selenium nanoparticles etc. could destroy the lysosomal membrane and destroy the lysosomal membrane permeabilization (LMP)^18^. As shown in Fig. 3B3, there was enrichment of red fluorescence observed in control MCF-7 cells, but after FA-UA-CS-NPs treatment, MFI of red fluorescence was significantly decreased, indicating the release of AO from ruptured lysosomes. The results demonstrated that FA-CS-UA-NPs could transport preferentially into lysosomes and then escape from lysosomes and entered into the cytoplasm after destructing the integrity of lysosomal membrane.

### Effects of FA-CS-UA-NPs on mitochondria

Next interesting questions would be what would happen and whether FA-CS-UA-NPs could attack and enter into the mitochondria. To address these, Mito Tracker (green color) was used as a mitochondrial fluorescence probe to detect the spatial relationship between FA-CS-UA-NPs and mitochondria in MCF-7 cells. There was only a little overlap of red and green color after 10 min and 30 min incubation. However, appreciable overlay of red and green color could be observed after incubation for 60 min (Fig. 3C1), which was also proved by the cross line analysis (Fig. 3C2). These results suggested FA-CS-UA-NPs entered into mitochondria after being released from lysosomes.

Mitochondria, the energy factories of cells, generate most of ATP that used as a source of chemical energy for cell metabolism. Mitochondria are involved in vital processes of cells, such as molecular signal transduction, cellular differentiation, growth and cell death, etc. As shown in Fig. 3C3, there was a significant decrease in mitochondrial membrane potential (ΔΨm) in MCF-7 cells harboring FA-CS-UA-NPs, implying that FA-CS-UA-NPs could destruct the mitochondrial membrane integrity and induce the collapse of ΔΨm in MCF-7 cells.

### Growth inhibition and apoptosis/necrosis in MCF-7 cells induced by FA-CS-UA-NPs

**Figure 4:**
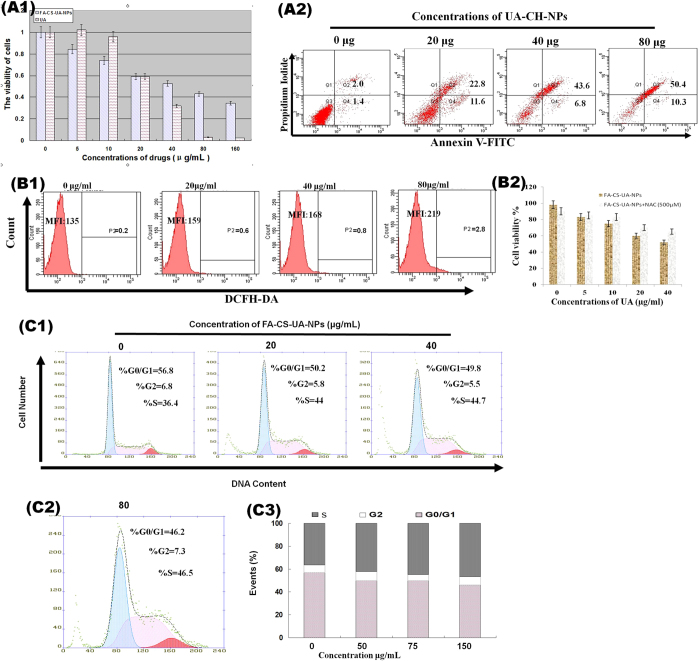
Effects of FA-CS-UA-NPs on cancer cell viability. (A1) Showed comparative viability frequencies of MCF-7 cells treated for 48 h with different concentrations of UA (hatch bars) and FA-CS-UA-NPs (gray bars). Note that at doses of 40–120 μg/mL, FA-CS-UA-NPs induces significantly greater killing effects on MCF-7 cells than UA (P < 0.05 for comparisons between UA and FA-CS-UA-NPs groups). (A2) Showed representative flow cytometry histograms indicating dose-dependent increases in Annexin V-stained early apoptosis and PI-stained necrosis (late apoptosis) rates of MCF-7 cells induced after 48-hr treatment with different doses of FA-CS-UA-NPs. (B1) Showed that ROS generation in cells was indicated by MFI of DCFH-DA (fluorescent indicator of ROS). (B2) Showed that mean cell viability frequencies of MTT assay evaluating the effects of NAC on cell viability of MCF-7 cells. Cells were pretreated with 500 μM NAC for 4 h and then exposed for 24 h to FA-CS-UA-NPs. Note that NAC could block ROS production leading to reduction in FA-CS-UA-NPs-mediated killing. (C1–C3) showed the cell cycle alterations in MCF-7 cells treated for 24 h with different concentrations of FA-CS-UA-NPs. Note a dose-dependent S phase arrest.

MTT assay was performed to detect the cell viability of MCF-7 cells after 48 h treatment with different concentrations of UA and FA-CS-UA-NPs, respectively. As shown in Fig. 4A1, at doses of 40–120 μg/mL, FA-CS-UA-NPs displayed stronger killing effects on MCF-7 cells than UA. For further detection of the death mode of MCF-7, MCF-7 cells were incubated with different concentrations of FA-CS-UA-NPs, and then co-stained by Annexin V-FITC and PI and measured by flow cytometry. Figure 4A2 showed that early apoptosis and necrosis(late apoptosis) appeared to represent the major death mode of MCF-7 cells induced by FA-CS-UA-NPs.

### FA-UA-CS-NPs induced ROS overproduction and cell cycle arrest

The intracellular ROS level in MCF-7 cells upon engulfing FA-CS-UA-NPs was investigated by MFI of DCFH. DCFH is formed through intracellular esterase hydrolyzation of a cell absorbed inflorescent probe (DCFH-DA). Figure 4B1 showed that with increasing concentrations of FA-CS-UA-NPs, MFI of DCFH in MCF-7 cells increased significantly, demonstrating the up-regulation of intracellular ROS level upon FA-CS-UA-NPs exposure. Moreover, when we detected the level of ROS generation induced after the different times of FA-CS-UA-NPs treatment, we found that the ROS generation was increased upon FA-CS-UA-NPs exposure from 4 to 12 h, but decreased at the time from 12 to 24 h of FA-CS-UA-NPs exposure (Figure S5). These results implied that the apoptotic events initiated after FA-CS-UA-NPs treatment for 12 h. To further examine the contribution of ROS generation to FA-CS-UA-NPs-induced apoptosis, MCF-7 cells were co-cultured with FA-CS-UA-NPs and N-acetyl-L-cysteine (NAC), the ROS inhibitor, and then assessed for the viability using MTT assay. The results showed that the addition of NAC significantly reduced the ability of FA-CS-UA-NPs to inhibit cellular viability of MCF-7 cells (Fig. 4B2). FA-CS-UA-NPs-induced death in MCF-7 cells appeared to correlate with ROS production (Fig. 4B1).

In parallel, the flow cytometry analysis of PI staining was used to determine the changes in cell cycle distribution induced by FA-CS-UA-NPs. Figure 4C3 showed MCF-7 cells were arrested in S phase, and the cell sub-population in S phase remarkably increased over the increases in the concentrations of FA-CS-UA-NPs (Fig. 4C1–C3). These results suggested that FA-CS-UA-NPs arrested or accumulated MCF-7 cells in DNA synthesis phase, blocked advancing into mitosis phase and ultimately led to the irreversible apoptosis.

### FA-CS-UA-NPs-induced anti-cancer activities *in vivo*

**Figure 5:**
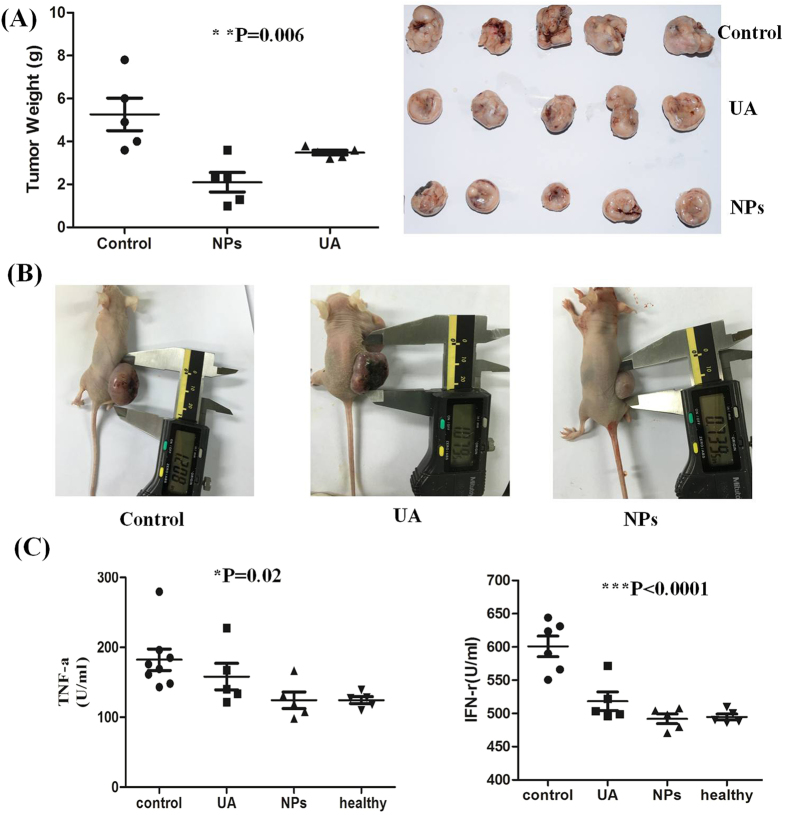
Anti-cancer efficacy of FA-CS-UA-NPs in MCF-7 nude mouse model. (**A**) Mean weight and images of tumors after i.p. administrated with 0.2 ml of 0.9% physiological saline (Control group), 12.5 mg/kg b.w. ursolic acid (UA group) and 12.5 mg/kg b.w. FA-CS-UA-NPs (NPs group). (**B**) Representative images of tumor sizes in MCF-7 xenograft mice. (**C**) ELISA data of serum cytokines (TNF-αand INF-r) in different treated groups. P values indicating statistical significance are derived from ANOVA analysis of control, UA and UA-NPs groups. Nonparametric t test shows similar levels of significance.

The anti-cancer effects of FA-CS-UA-NPs were determined in MCF-7 cancer xenograft mice. Female Balb/C mice were injected with MCF-7 cells, and at 10 days after injection, the mice were randomly divided into three groups. The control group, positive control group and test group were intraperitoneal injection (i.p.)/day with 0.2 ml of physiological saline, and 12.5 mg/kg bw of UA and FA-CS-UA-NPs, respectively. Mice were sacrificed and evaluated for changes in cancer burdens after the 8^th^ treatment. As shown in Fig. 5A,B the FA-CS-UA-NPs-treated group exhibited significant decreases in the tumor burden. The tumor weight in FA-CS-UA-NPs-treated group was 2.1 ± 1.02 g, which was significantly lower than those of UA and saline control groups (5.26 ± 1.69 g, p < 0.05), and UA-treated group (3.48 ± 0.24 g) (P < 0.005).

Figure 5C showed the statistical data of cytokines (IFN-γ and TNF-α) in serum detected by ELISA using ANOVA analysis. After treated with UA or NPs, there was an obvious decrease in expression of IFN-γ and TNF-α comparing with saline control group, implying drug-treated group could modify the immune system of tumor-burdened mouse into a good trend.

## Discussion

In the current study, we develop a kind of novel FA-CS-UA-NPs to increase UA solubility, selectively target cancer cells and achieve better therapeutic efficacy against breast cancers in both *in vitro* and *in vivo* models. Several studies have assessed high doses of UA for the anti-tumor effects in mouse model. Shanmugam, *et al*.^19^ reported that 6-week high-dose UA treatment regimen (200 mg/kg b.w./every day) inhibited the growth of DU145 cells in nude mice. Hursting, *et al*.^20^ reported UA at 106 mg/kg b.w./day for 3 weeks was effective against tumor growth in a model of postmenopausal breast cancer. Similarly, Saraswati, *et al*.^21^ showed that daily treatments with UA at 100 mg/kg b.w./day dose for 30 days significantly inhibited tumor growth or cell viability in both ascites and solid tumor models. In contrast, our therapeutic UA dose by using the novel FA-CS-UA-NPs is 10 times lower than those reported doses. Such reduced dose and duration for FA-CS-UA-NPs (12.5 mgUA/Kg b.w./day for 10 days) significantly inhibit tumor growth and reduce tumor weights better than UA alone in MCF-7 xenograft nude mouse model. The dramatic improvements for dosing and duration are attributed to our innovative chitosan/folate nano-carrier system that is able to efficiently carry or deliver UA and selectively target cancer cells, suggesting that FA-CS-UA-NPs delivering UA may significantly reduce or diminish side effects or toxicities potentially induced by high doses of UA or other drugs. Moreover, data of serum cytokines showed that treatments with FA-CS-UA-NPs do not induce apparent pro-inflammatory responses or tissue damages.

The enhanced UA efficacy by using FA-CS-UA-NPs might be explained by two main aspects. First, FA-CS-UA-NPs appear to harness UA pharmacological potency by means of an increased solubility of UA and folate-directed on-target effect. As shown by the MTT results (Fig. 4), FA-CS-UA-NPs more dramatically inhibit MCF-7 cancer cell growth or viability in MCF-7 cancer cells than UA alone. Second, the augmented efficacy of FA-CS-UA-NPs versus UA may also be attributed to the enhanced permeability and retention (EPR) effect, a unique tumors-related phenomenon that can be targeted as a basis for development of macromolecular anti-cancer therapy. Because numerous holes or gaps with 50~500 nanometers exist between endothelial cells in tumor blood vessels and tumor tissues, drug-carriers or macromolecular drugs with sizes of 50~500 nm show selective extravasation and retention and result in prominent anti-tumor effects^22^. Our FA-CS-UA-NPs display sizes about 100–200 nm, and therefore may easily target cancer tissues/cells thanks to the EPR effect in tumor tissues/vessels. FA-CS-UA-NPs with such unique sizes can also enjoy longer half-lives as they are not easily phagocytosed or cleared by macrophages^23^.

FA-CS-UA-NPs appear to have the aggregation-resistant properties in suspension as the high absolute value of zeta potential (>25 mV) has been reported to produce big repulsive electrostatic force between nanoparticles avoiding aggregation. The zeta potential of FA-CS-UA-NPs is + 39.3 mV, suggesting that they are stable and could be easily internalized by tumor cells. In addition, due to electrostatic interactions, positively charged FA-CS-UA-NPs can be more easily internalized by cancer cells whose membrane is negatively charged^24^,^25^.

**Figure 6:**
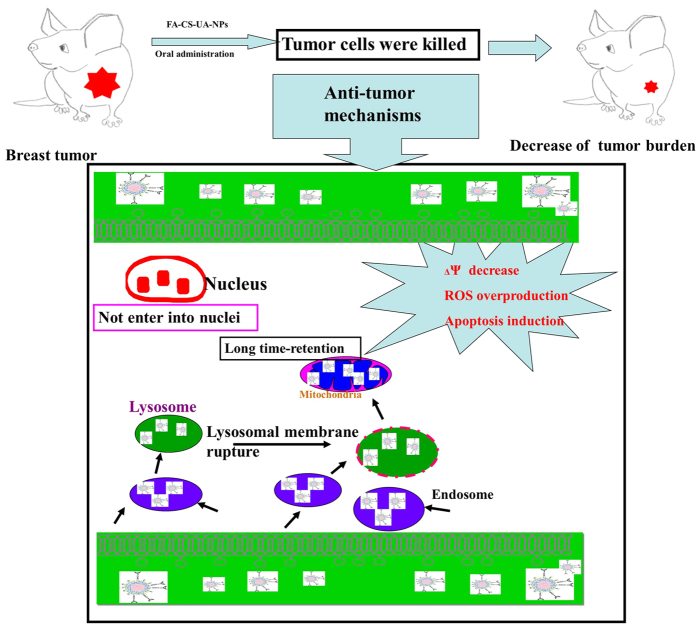
Proposed killing mechanisms of MCF-7 cells induced by FA-CS-UA-NPs.

The current study also provides potential intracellular trafficking mechanisms underlying FA-CS-UA-NPs-mediated killing of cancer cells (Fig. 6). FA-CS-UA-NPs target and enter tumor cells via a folate receptor-mediated endocytosis pathway, which is in energy- and time-dependent fashions. Upon being internalized in lysosomes, FA-CS-UA-NPs appear to destruct permeability and integrity of lysosomal membrane and thereafter get released to the cytoplasm. Then, FA-CS-UA-NPs can enter into mitochondria and induce the overproduction of ROS (Fig. 4B). It has been widely reported that nanomaterials can induce the overproduction of ROS in cancer cells, and such ROS overproduction is responsible for the anti-cancer effect and toxicity of nanomaterials. As a natural byproduct of the normal metabolism of oxygen, ROS has important roles in cell signaling and homeostasis^26^. However, dramatic increases of ROS in cells would induce the lipid peroxidation, protein oxidation, mitochondrial damages and other cytotoxic effects, which ultimately led to the collapse of Δψ and even cellular apoptosis. On the other hand, FA-CS-UA-NPs treatments of cancer cells also lead to cell cycle arrest and prevention from entering mitosis phase for cell replication (Fig. 4C). This is consistent with earlier reports suggesting that nano-based materials can cause the DNA damage and chromosomal aberrations and disturbance of cell cycle in the process of cell division^27^.

In summary, we have synthesized a novel nano-carrier using biodegradable chitosan to encapsulate the anticancer drug UA for enhanced water solubility, with folate loaded on surface to improve the UA’s targeting and anti-cancer activity. We show that the synthesized nano-drugs, FA-CS-UA-NPs, can stably target cancer cells, induce appreciable anti-cancer activities in breast cancer cells with underlying mechanisms, and confer detectable therapeutic effects against breast cancer *in vivo*, with no or very low side effects.

## Materials

All chemicals were of analytical grade. UA (98.6%) and chitosan (M = 1,600) were purchased from Shanxi Huike Botanical Development Co., Ltd. (China) and Jinan Haidebei Marine Bioengineering Co. Ltd. (China), respectively. Folate, rhodamine B, Ethyl-(3-3-dimethylam -inopropyl) carbondiimide hydrochloride (EDC), N-hydroxy-succinimide (NHS), 3-(4,5)-dimethylthiahiazo(-z-y1)-3,5-di-phenytetrazoliumromide (MTT), N-acetylcysteine (NAC ) were purchased from sigma (USA). Propidium Iodide (PI), Annexin V/PI apoptosis detection kit, 2′,7′-dichlorfluorescein-diacetate(DCFH-DA), 2-(4-Amidinophenyl)-6-indolecarbamidine dihydo-chloride (DAPI), 2-(6-Amino-3-imino-3H-xanthen-9-yl) benzoic acid methyl ester (Rhodamine123), caspase3, caspase8 and caspase9 activity assay kit, Mito-Tracker green, Lyso-Tracker red were purchased from Beyotime institute of biotechnology (China). RPMI-1640 medium, DMEM medium, fetal bovine serum (FBS), trypsin and penicillin-streptomycin were purchased from Gibco BRL (USA). Matrigel was obtained from BD biosciences. Milli-Q water was used in all experimental processes. All experimental protocols were approved by IACUC and IBC in accordance with the guidelines of department of Chemistry, the first affiliated hospital of Jinan University, and UI college of Medicine.

### Preparation of CS-UA-NPs

32 mg of chitosan was dissolved in 5 ml of 1% (v/v) glacial acetic acid. 10 mg UA, 30 mg EDC and 8 mg NHS were dissolved in 5 ml of THF, after that it was added to the above chitosan solution. Then, the solution was kept on constant magnetic stirring overnight at room temperature. After precipitation with pure water, the solution was centrifuged twice at 11,000 rpm for 20 min to remove excess amounts of THF and unencapsulated UA. Finally, NPs were lyophilized for 48 hours using lyophilizer for storage in powdered form. To determine the NPs uptake by cells, NPs containing a fluorescent dye (rhodamine B) were prepared using the above procedure, except that 250 μg dye was added with the addition of UA to the chitosan solution. The incorporated dye acts as a probe for NPs and offers a sensitive method to determine qualitatively and quantitatively intracellular binding including eventual uptake and retention.

To improve the cell-specific targeting of NPs by cancer cells, the NPs were conjugated with folate to target folate receptor bearing tumor cells. Briefly, the solution of NPs was mixed with folic acid (0.5 g/L) for 3 h at 4 °C, and then centrifuged at 3,000 rpm for 15 min to remove the unbinded folate.

### Characterization of CS-UA-NPs

The size distribution and zeta potential of the nanoparticles was estimated using a Zetasizer Nano ZS (Malvern Instruments, UK). TEM (Philips Co, Holland) and AFM (Veeco, USA) were used to characterize the morphology and dispersed state of the nanoparticles. The micro-graphs were obtained on Hitachi (H-7650) for TEM operated at an accelerating voltage at 80 kV. AFM images were achieved by tapping mode in air. A scanning spectro-photometer (Varian, USA) with a 1-cm path length was employed to measure the UV/VIS absorbance of as-prepared products.

To measure the drug (UA) loading rate of FA-CS-UA-NPs, 10 mg lyophilized nanoparticles were dissolved in 1 ml of methanol, and then the amount of UA in the solution was determined by High Pressure Liquid Chromatography (HPLC). HPLC detection was performed using a C18 column (5 μm, 250 mm × 4.6 mm), whereas the mobile phase, consisting of methanol and 0.1% acetic acid (88:12) (v/v), was maintained at a flow rate of 1.0 mL/minute. The ultraviolet detector wavelength was 215 nm and the injection volume was 20 μL. The drug loading was calculated according to the formula: loading rate % = UA/(CS + UA) × 100%.

To detect the *in vitro* release rate of Rh B from the NPs, 10 ml of 1 mg/ml of RhB-loaded NPs were shaken in water bath at 37 °C at different time points. At each time point, 300 μl of samples were taken out and centrifuged. 20 μl of supernatant was collected and measured for Rh B concentration in HPLC. HPLC detection was performed using a C18 column (5 μm, 250 mm × 4.6 mm), whereas the mobile phase, consisting of methanol and 0.1% acetic acid (70:30) (v/v), was maintained at a flow rate of 1.0 mL/minute. The ultraviolet detector wavelength was 548 nm of excitation and 578 nm of emission. Release rate was calculated through dividing supernatant RhB by total RhB. The release rates of RhB from NPs were <4% and <10% in 4 and 12 hours, respectively. Figure S8 showed the release of Rhodamine B from the nanoparticles after stirring for different time periods at 37 °C water bath.

### Cell Lines and Cell Culture

MCF-7 breast cancer cells and Colo205 colon adenocarcinoma cells were purchased from American Type Culture Collection (ATCC, USA). All cell lines were grown in DMEM media supplemented with fetal bovine serum (10%), penicillin (100 units/mL), and streptomycin (100 units/m L) at 37 °C in a humidified incubator with 5% CO_2_ atmosphere.

### Cellular Uptake of FA-CS-UA-NPs

The cellular uptake of FA-CS-UA-NPs was qualitatively detected by fluorescence microscopy. Briefly, treated cells cultured on cover glass in 6-well plates until 70% confluence were incubated with different concentrations of rhodamine B-loaded FA-CS-UA-NPs for various periods of time. The cells were then washed three times by PBS and examined under a fluorescence microscope (Nikon, Japan).

Flow cytometry was used to quantify the cellar uptake of FA-CS-UA-NPs in MCF-7 cells. Briefly, 2 × 10^5^ cells/well were plated on 6 well plates and incubated 24 h for attachment. After that, the cells were incubated with designated concentrations of rhodamine B-loaded NPs for different periods. Before analyzed by flow cytometry (BD Inc, USA), the cells were washed triple with PBS and detached by trypsin, washed twice with PBS. The mean fluorescence intensity (MFI) was the indicator of uptake of NPs by cells.

MCF-7 cells (10^6^ cells/ml) were cultured with 2 μg/ml (terminal concentration) UA or FA-CS-UA-NPs for 24 h. Then, the cells were washed three times using cold PBS with the supernatants discarded, and then added to the mobile phase, consisting of methanol and 0.1% acetic acid (88:12) (v/v) for HPLC detection. The flow rate was 1.0 mL/minute. The ultraviolet detector wavelength was 215 nm and the injection volume was 20 μL.

### MTT assay

Cell viability was determined by measuring the ability of cells to transform MTT to a purple formazan dye. Cells were seeded in 96-well culture plates at 5 × 10^3^ cells/well for 24 h. The cells were then incubated with FA-CS-UA-NPs at different concentrations for different periods of time. After treatment, 20 μL/well of MTT solution (5 mg/mL PBS) was added to the well and incubated for another 4 h. For further confirmation the effects of ROS on cell apoptosis, the ROS inhibitor- N-acetyl-L-cysteine (NAC) (2.5 mM), were added into cells 30 min prior to adding drugs, and then performed MTT assay.

### Cell cycle analysis by flow cytometry

MCF-7 cells with or without treatment with FA-CS-UA-NPs for 24 h, were trypsinized, washed twice with PBS, and then, fixed with 70% chilled ethanol overnight. The fixed cells were washed twice with PBS, treated with RNase A, stained with PI (50 μg/mL), and analyzed by flow cytometer.

### Cell apoptosis determination

An Annexin V-FITC/PI apoptosis detection kit was used to detect the apoptotic cells according to the manufacturer’s instructions. After treatment with different concentrations of FA-CS-UA-NPs, MCF-7 cells were harvested and washed with cold PBS and suspended in 500 μl Annexin V binding buffer. After incubation with 5 μl FITC labeled-Annexin V and 5 μl PI at room temperature in the dark, the samples were immediately analyzed by flow cytometer at 488 nm.

### Evaluation of mitochondrial membrane potential (ΔΨm) and intracellular reactive oxygen species (ROS) generation

To detect the changes in ΔΨm and ROS levels, the MCF-7 cells were treated with different concentrations of FA-CS-UA-NPs and then harvested to incubate with rhodamin123 and DCFH -DA for 30 min in dark at room temperature, separately. The cells were collected and washed twice with PBS. The resulting fluorescence was measured by flow cytometer both excited by 488 nm.

### *In vivo* xenograft mouse model

#### Mice model

MCF-7 xenograft model was established using BALB/c nude mice of 4~6 weeks age. The animals were obtained from Beijing HFK Bioscience Co., LTD and quality checks were supervised by Institute of Laboratory of Animal Science (CAM&PUMC). MCF-7 cells (10^7^ cells in 50 μL serum free medium) with 50 BALB/c nude mouse of 4~6 weeks age matrigel were injected into the back of mouse. The mouse were i.p. administrated with physiological saline, ursolic acid (12.5 mg/kgb.w./day), FA-CS-UA-NPs (12.5 mg/kg b.w./day) for nine times. One group of normal healthy mice were used as negative control. There was 5 mice in each group and no mice dead during the whole experimental process.

All experimental protocols were approved by Animal Ethics Committee of Guangdong province, China. And all the experiments were performed in accordance with relevant guidelines and regulations of Animal Ethics Committee of Guangdong province, China.

### Measurement of cytokines in blood

The blood of each mouse was collected through the orbital venous, then the serum was collected by centrifugation at 3000 rpm/min for 10 min. And the serum levels of cytokines (Interferons IFN-γ, tumor necrosis factor TNF-α) were determined by ELISA according to the manual instruction of cytokines ELISA kit from R&D Systems.

### Statistical analysis

Statistical analysis was performed using nonparametric t test or ANOVA test, with *P < 0.05 regarded as statistically significant.

## Additional Information

**How to cite this article**: Jin, H. *et al*. Folate-Chitosan Nanoparticles Loaded with Ursolic Acid Confer Anti-Breast Cancer Activities *in vitro* and *in vivo*. *Sci. Rep.* **6**, 30782; doi: 10.1038/srep30782 (2016).

## Supplementary Material

Supplementary Information
