# Supplementary material for: Folate-Chitosan Nanoparticles Loaded with Ursolic Acid Confer Anti-Breast Cancer Activities in vitro and in vivo
Source: Sci Rep. 2016 Jul 29;6:30782. doi: 10.1038/srep30782 (PMC4965748; doi:10.1038/srep30782)
Supplement: Supplementary Information [file srep30782-s1.doc]

**Folate-Chitosan Nanoparticles Loaded with Ursolic Acid Confer Anti-Breast Cancer Activities in vitro and in vivo.**

**Folate-Chitosan Nanoparticles Loaded with Ursolic Acid Confer Anti-Breast Cancer Activities in vitro and in vivo**

Hua Jin^1,2^, Jiang Pi^1^, Fen Yang^1^, Jinhuan Jiang^1^, Xiaoping Wang^3^, Haihua Bai^4^, Mingtao Shao^3^, Lei Huang^5^,Haiyan Zhu^4^, Peihui Yang^4^, Lihua Li^4^, Ting Li^1^, Jiye Cai^1,4^*, and Zheng W. Chen^2^*

^1^ State Key Laboratory of Quality Research in Chinese Medicines, Macau University of Science and Technology, Macau, 999078, China

^2^ Department of Microbiology and Immunology, University of Illinois, Chicago 60612, USA

^3^ the First Affiliated Hospital of Jinan University, Guangzhou 510632, China

^4^Department of Chemistry, materials science and engineering, Jinan University, Guangzhou 510632, China

^5^Treatment and Research Center of Infectious Diseases, the 302 Hospital of PLA, Beijing, 100039, China

^*^**Corresponding author:**

**Jiye Cai** E-mail: [tjycai@jnu.edu.cn](mailto:tjycai@jnu.edu.cn)

**Zheng W. Chen E-mail:** zchen@uic.edu

Supplemental data


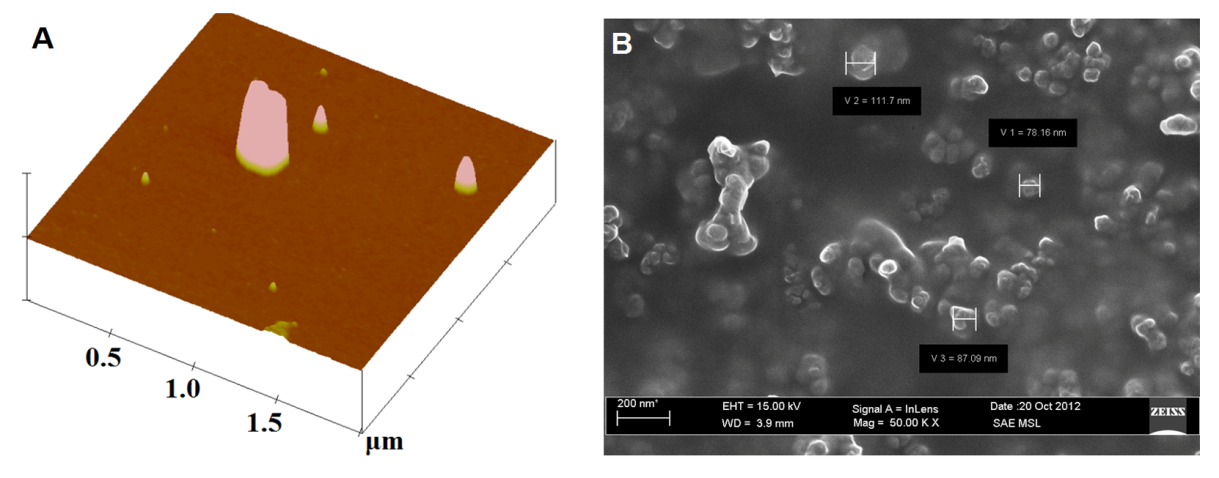


Fig.S1 The morphology of FA- CH-UA NPs. (A) and (B) are AFM and TEM images of FA- CH-UA NPs, respectively.


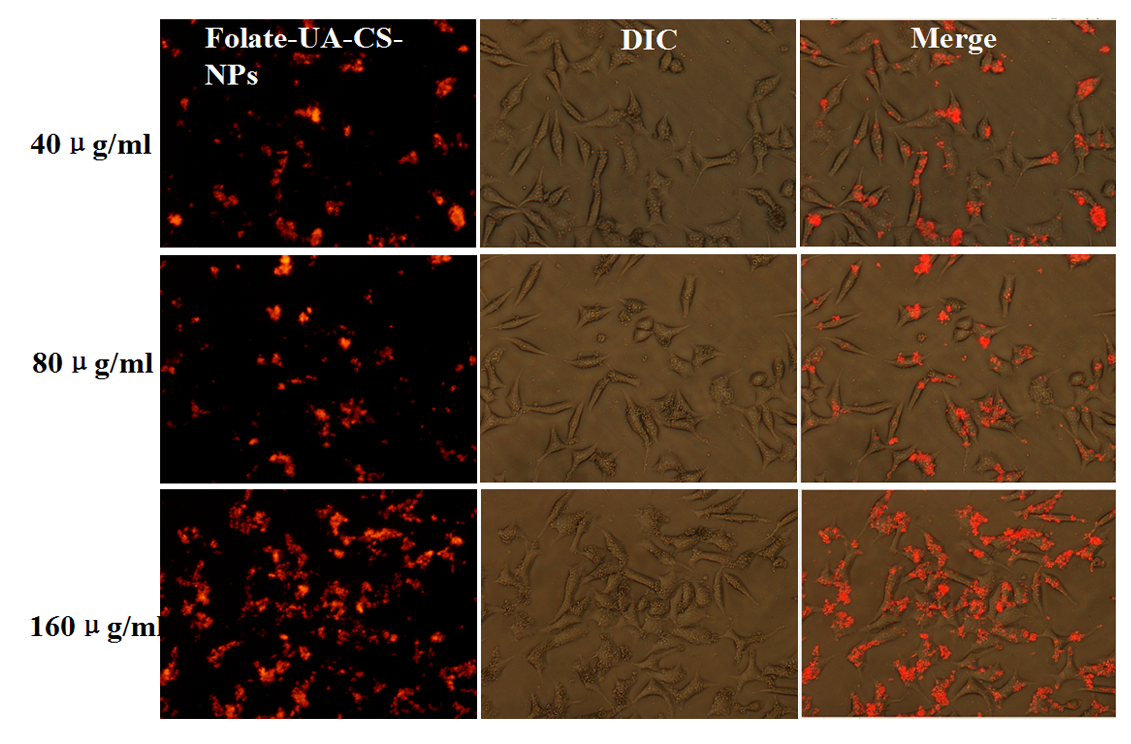


**Fig.S2** Different doses of FA-UA-CS-NPs were internalized by MCF-7 cells after cocultured for 3 hours at 37 ℃. Rhodamine B was loaded inside the NPs, therefore, the red fluorescence showed the location of NPs in/on cells.


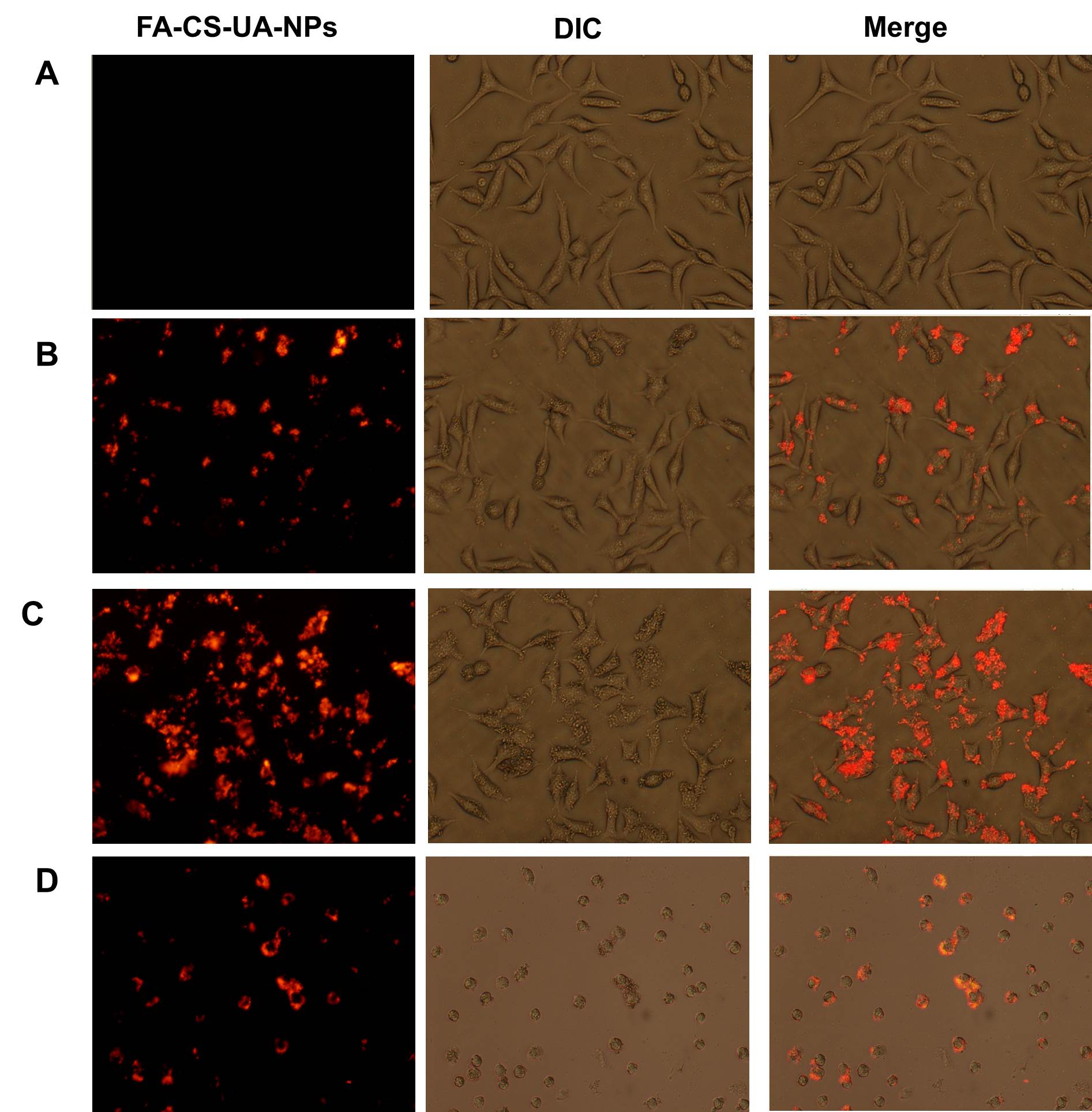


Fig.S3 Determination of internalization of FA-UA-CS-NPs by MCF-7 cells induced by different cocultures periods. Fluorescence images of MCF-7 cells treated with 40 μg/mL of rhodamine B loaded FA-UA-CS-NPs for 0 (A), 2 (B), 24 (C), 96 h (D), respectively.


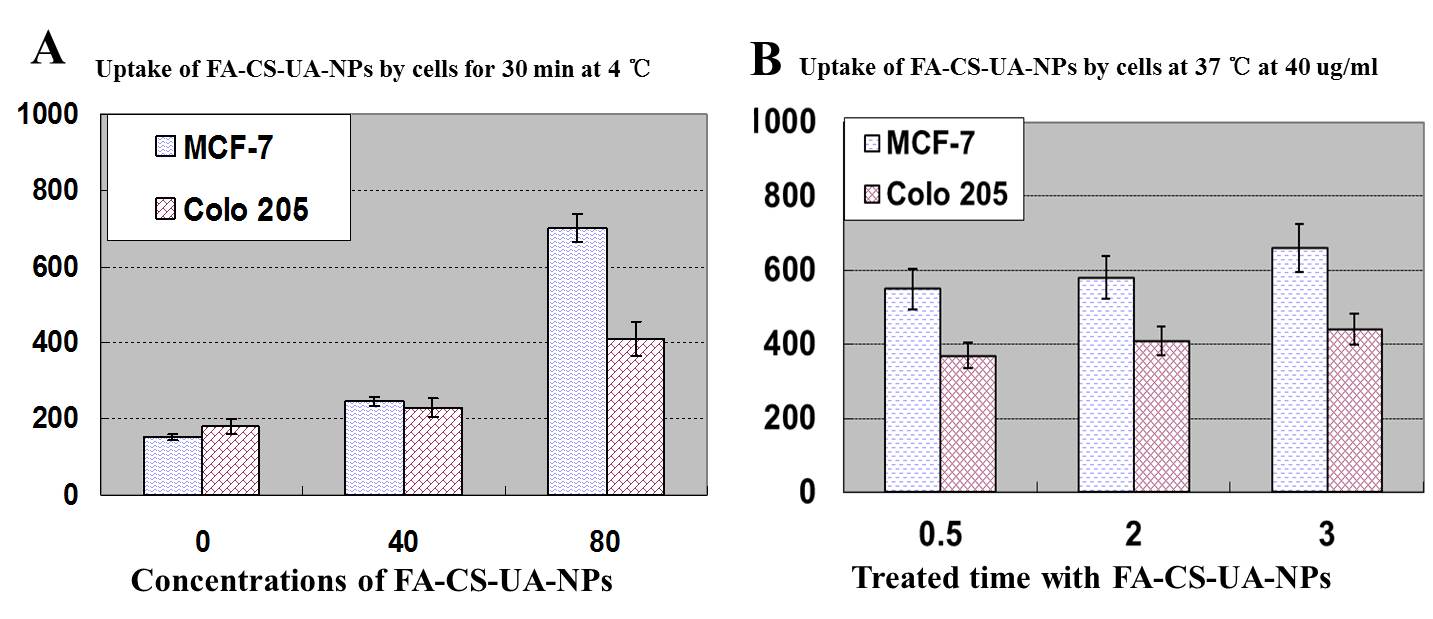


Fig.S4 To determine the effects of cell types and energy on cellular uptake of FA-UA-CS-NPs. The uptake of rhodamine B loaded FA-UA-CS-NPs by MCF-7 cells and colo 205 cells at 4 ℃ (A)and 37 ℃(B), respectively. The mean fluorescence intensity (MFI) is the indicator of FA-UA-CS-NPs uptaken by cells. Flow cytometry with excitation and emission wavelengths was set at 540 and 625 nm, respectively.


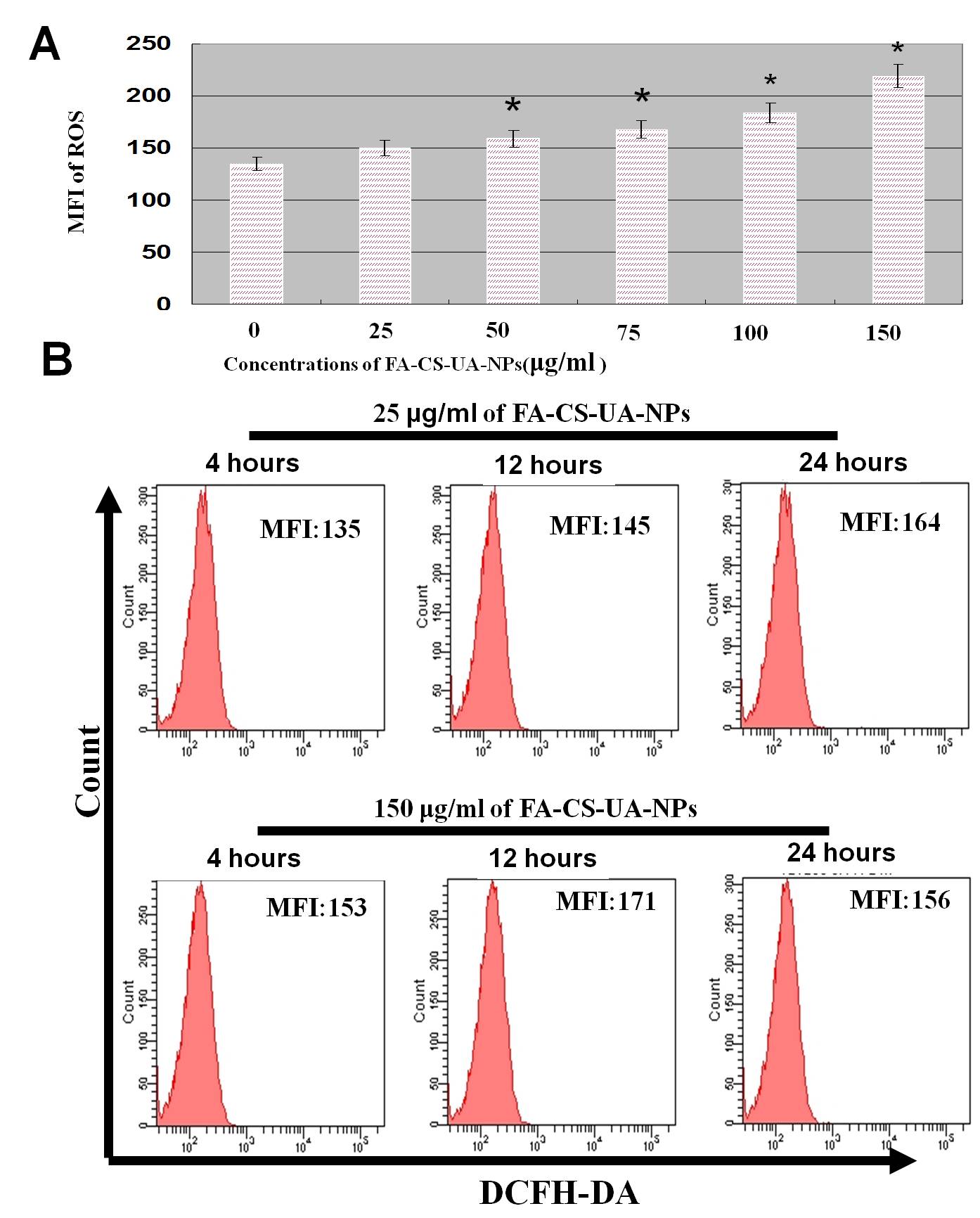


Fig.S5 To determine the effects of doses and coculture periods of FA-CS-UA-NPs on ROS production in cells. Flow cytometric analysis of ROS production in MCF-7 cells treated with low concentration (25 μg/ml) and high concentration (150 μg/ml) of FA-CS-UA-NPs for different time. *P<0.05


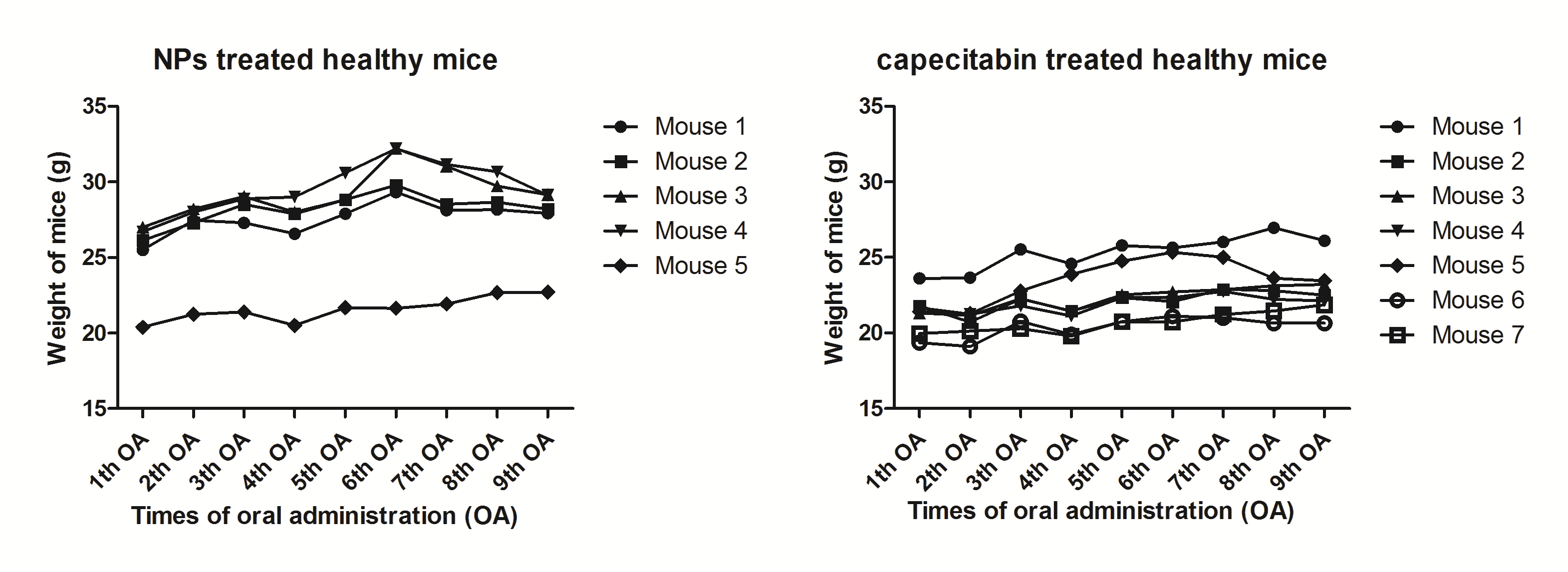


Fig.S6 To determine the toxic effects of FA-CS-UA-NPs on healthy mice. The body weight was measured after the mice were oral administrated (OA) with FA-CS-UA-NPs and capecitabin for 9 times (19 days). The doses were the same as in treated mice, i.g. 12.5 mg/kg b.w. ever two days, 100 mg/kg b.w. Every two days. It can be seen that the mouse body weight in NPs treated group was significantly higher than that of capecitabin treated group.


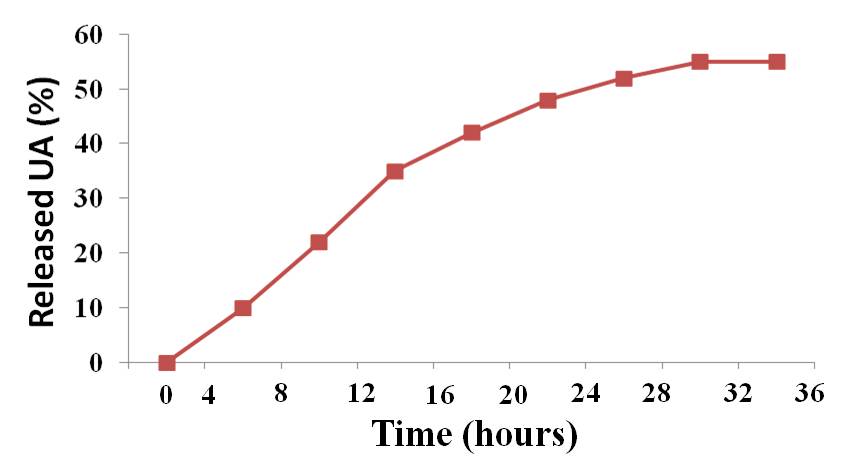


Fig.S7 In vitro release profiles of UA from FA-CS-UA NPs in PBS (0.01M, PH=7.4). The concentrations of UA were determined by HPLC.


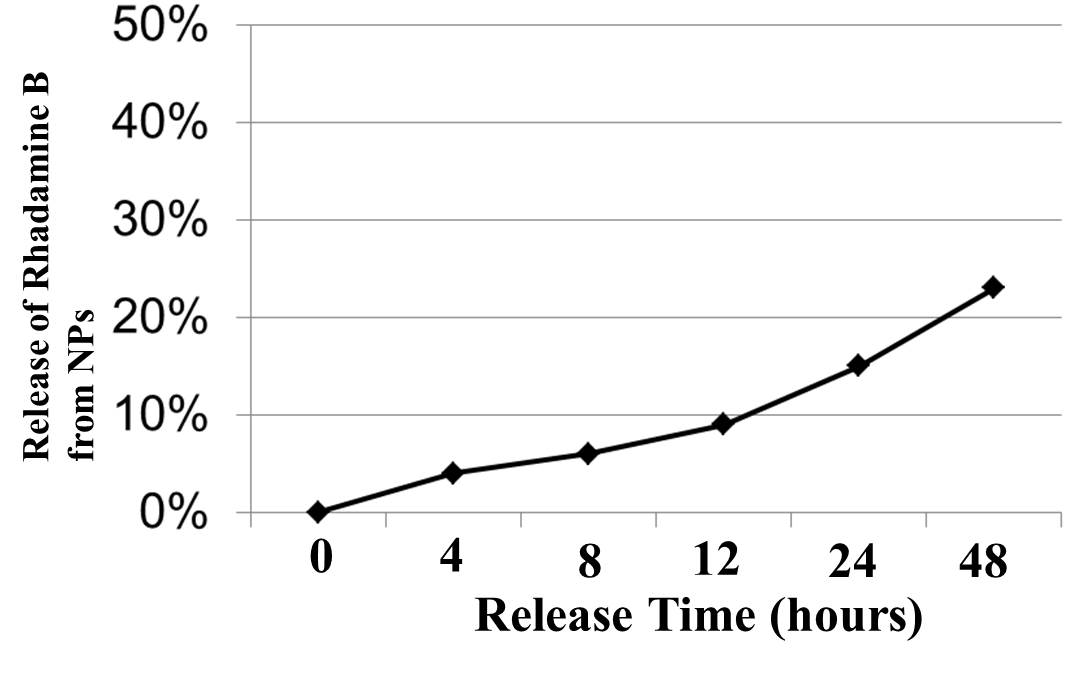


Fig. S8 Release rates of Rhodamine B from the nanoparticles after stirring for different time points at 37 ℃ stirring water bath.
